# Scleral buckling versus vitrectomy for macula-off rhegmatogenous retinal detachment as accessed with spectral-domain optical coherence tomography: a retrospective observational case series

**DOI:** 10.1186/1471-2415-13-12

**Published:** 2013-04-15

**Authors:** Chunmei Huang, Te Fu, Tonghe Zhang, Xinyi Wu, Qiang Ji, Ruili Tan

**Affiliations:** 1 Department of Ophthalmology, Qilu Hospital of Shandong University, 107# Wenhua Xi Road, Jinan 250012, People's Republic of China; 2 Department of Retina and Vitreous, The Second People's Hospital of Jinan, 148# Jingyi Road, Jinan, 250001, People's Republic of China

**Keywords:** Optical coherence tomography, Retinal detachment, Sclera buckling, Vitrectomy surgery, Subretinal fluid

## Abstract

**Background:**

Scleral buckling surgery and pars plana vitrectomy are competing methods in the treatment of retinal detachment. The recent development of spectral-domain optical coherence tomography (SD-OCT) has dramatically improved the visualization of the photoreceptor layer relative to conventional OCT, and offers new opportunities to investigate the discordances between anatomic and functional outcomes after retinal detachment surgery. Hence, the study aim was to use SD-OCT to compare the postoperative macular recovery between scleral buckling and vitrectomy for macular-off rhegmatogenous retinal detachment.

**Methods:**

In this retrospective observational case series, we observed 32 patients who underwent scleral buckling surgery (group 1) and 26 patients who underwent pars plana vitrectomy (group 2) as the primary surgery for macula-off rhegmatogenous retinal detachment. OCT was used to examine microstructural changes in the macular area.

**Results:**

The mean visual acuity improvement was 0.4 ± 0.8 logMAR in group 1 and 0.7 ± 0.9 logMAR in group 2. As detected by SD-OCT, subretinal fluid was present in 26 of the group 1 eyes (81.3%) and 5 of the group 2 eyes (19.2%) at 8 weeks postoperatively.

This difference was statistically significant (Fisher’s exact test, P < 0.05). Moreover, detection by SD-OCT revealed epiretinal membranes in 5 of the group 1 eyes (15.6%) and 11 of the group 2 eyes (42.3%), a difference that was statistically significant (Fisher’s exact test, P < 0.05).

**Conclusions:**

Macular recovery and the mean visual acuity differed between the 2 groups of patients. With the help of SD-OCT, we observed that subretinal fluids could persist for a relatively longer period after scleral buckling. Based on our results, we conclude that primary vitrectomy surgery is a better choice for macular recovery of the macula-off rhegmatogenous retinal detachment.

## Background

The treatment of patients with primary rhegmatogenous retinal detachment (RRD) has undergone considerable changes in recent decades. Since pars plana vitrectomy (PPV) was established in 1971 by Machemer et al. [1], scleral buckling and PPV have competed as methods in the treatment of retinal detachment. Numerous studies have been published recommending one of the two methods [2-12]. Scleral buckling remains the method of choice in uncomplicated retinal situations, i.e., single breaks and/or a limited retinal detachment. In contrast, PPV is indicated in complicated situations, i.e., vitreous hemorrhage/opacity, proliferative vitreoretinopathy (PV), or breaks at the posterior pole [13-22].

Poor visual acuity (VA) or metamorphopsia may persist over time after a successful operation for macula-off retinal detachment. Both preoperative and postoperative factors are generally considered to contribute to the recovery of VA after surgery for this type of retinal detachment. The pre-operative factors include VA, height and duration of the macular detachment, and vitreomacular traction, while post-operative factors include cystoid macular edema [23,24], epiretinal membranes, retinal folds, persistent subretinal fluid, retinal folds [25], and persistent foveal detachment [26,27].

Optical coherence tomography (OCT) is a noninvasive real-time system that can be used to investigate retinal structures [28,29]. The more recent development of spectral-domain OCT (SD-OCT) has led to a 50-fold higher data acquisition speed, which has reduced the occurrence of motion artifacts, and has dramatically improved the visualization of the photoreceptor layer by using an axial resolution of 4 to 6 μm [30]. Therefore, SD-OCT imaging offers new and interesting opportunities to better understand the discordances between anatomic and functional outcomes after retinal detachment surgery. In this study, we used SD-OCT to compare macular recovery between PPV and scleral buckling in the treatment of macula –off RRD.

## Methods

This study was a retrospective observational case series. The preoperative data included age, sex, VA [converted to the logarithm of the minimal angle of resolution (logMAR)], the duration of detachment and the height of the macular detachment, lens status, and the PV grade. The height of the macular detachment was measured using the retinal thickness mode in the SD-OCT software and was defined as the distance between the inner surface of the retinal pigment epithelium and the outer surface of the neurosensory retina at the fovea.

Fifty-eight consecutive patients (58 eyes) with macula-off RRD underwent surgical repair at department of Retina and Vitreous, the Second People's Hospital of Jinan, Shandong province, China, between February 2011 and February 2012 were reviewed. All the patients met the inclusion/exclusion criteria. All eyes had primary retinal detachment without age-related macular degeneration, cataract, vitreous hemorrhage, macular hole, trauma, macular edema, intraocular inflammation, glaucoma, or retinal vascular occlusive diseases. Patients with systemic diseases, such as diabetes or hypertension, were excluded. We divided the patients based on retinal tears. If the tears were more than 2 disk diameter, the retinal detachment range was big (more than half of the retina) and obvious traction existed, we choose PPV; If retinal tears were small and gathered, the retinal detachment range was small and no obvious traction existed, we choose SB. PV grade showed no significant difference in the two groups, being less than grade B in all the patients. In our study, 32 eyes (group 1) and 26 eyes (group 2) successfully underwent scleral buckling and PPV respectively. Informed consent was obtained from all patients included in the study and the study was approved by the ethical committee of the Second People's Hospital of Jinan, Shandong province, China.

In ppv group, perfluoropropane (C3F8) tamponade was used in the patients who had tears located in the upper retina. Four patients (4 eyes) underwent phacoemulsification to deal with peripheral retinal holes and retina degeneration or dry hole areas. These four eyes underwent oil implantation 3 months later. Both silicone oil and perfluoropropane (C3F8) filled eyes did not develop cataract at final follow-up. Meanwhile, no gas remained in the vitreous cavity 2 months after the operation for group 2 eyes in which gas tamponade was used.

All patients underwent pre- and postoperative best-corrected logMAR VA testing, slit-lamp biomicroscopy, and dilated fundus examination. High-resolution, three-dimensional SD-OCT (Cirrus HD-OCT 4000 Zeiss, Germany) examinations were performed for each eye. The SD-OCT measurements were made after pupil dilation (0.5% tropicamide), with the patient in the sitting position and the head placed on a chin rest. SD-OCT measurements were also obtained 2 weeks, 1 month, and 2 months after the operation.

Values are given as the frequency and percentage for qualitative parameters, while the mean and standard deviation are provided for quantitative parameters. The best-corrected VA (BCVA) was evaluated before the operation and at the final follow-up using a paired *t*-test. The incidence of epiretinal membranes, subretinal fluid, discontinued external limiting membrane (ELM), and disruptions of the inner segment/outer segment junction and ELM were evaluated with Fisher’s exact test. The results were considered statistically significant at P < 0.05. Statistical analyses were performed using SPSS 11.0 software (SPSS Inc., Chicago, IL, USA).

## Results

Patient characteristics are shown in Table 1. The mean age of groups 1 (scleral buckling) and 2 (vitrectomy) were 36.38 ± 13.32 years (range, 17–59 years) and 44.69 ± 14.37 years (range, 19–67 years), respectively. The age, sex, duration of macular detachment (group 1: 12 ± 4.5 days; group 2: 15 ± 6.5 days), and the height of the macular detachment (group 1: 375 ± 107 μm; group 2: 470 ± 125 μm) showed no significant differences between the 2 groups.

**Table 1:** Preoperative characteristics of patients in the scleral buckling and vitrectomy groups

**Variable**	**Scleral buckling**	**Vitrectomy**	***P *value**
	**(n = 32)**	**(n = 26)**	
**Age**, mean ± SD	36.4 ± 13.3	44.7 ± 14.4	0.37**
(range)	17–59	19–67	
**Sex**			
Males	18	14	1.0*
Females	14	12	
**Visual acuity (X, logMAR),**			
mean ± SD (logMAR)	1.2 ± 0.9	1.3 ± 1.0	0.40**

* Squared test.

** Mann–Whitney *U* test.

**Figure 1:**
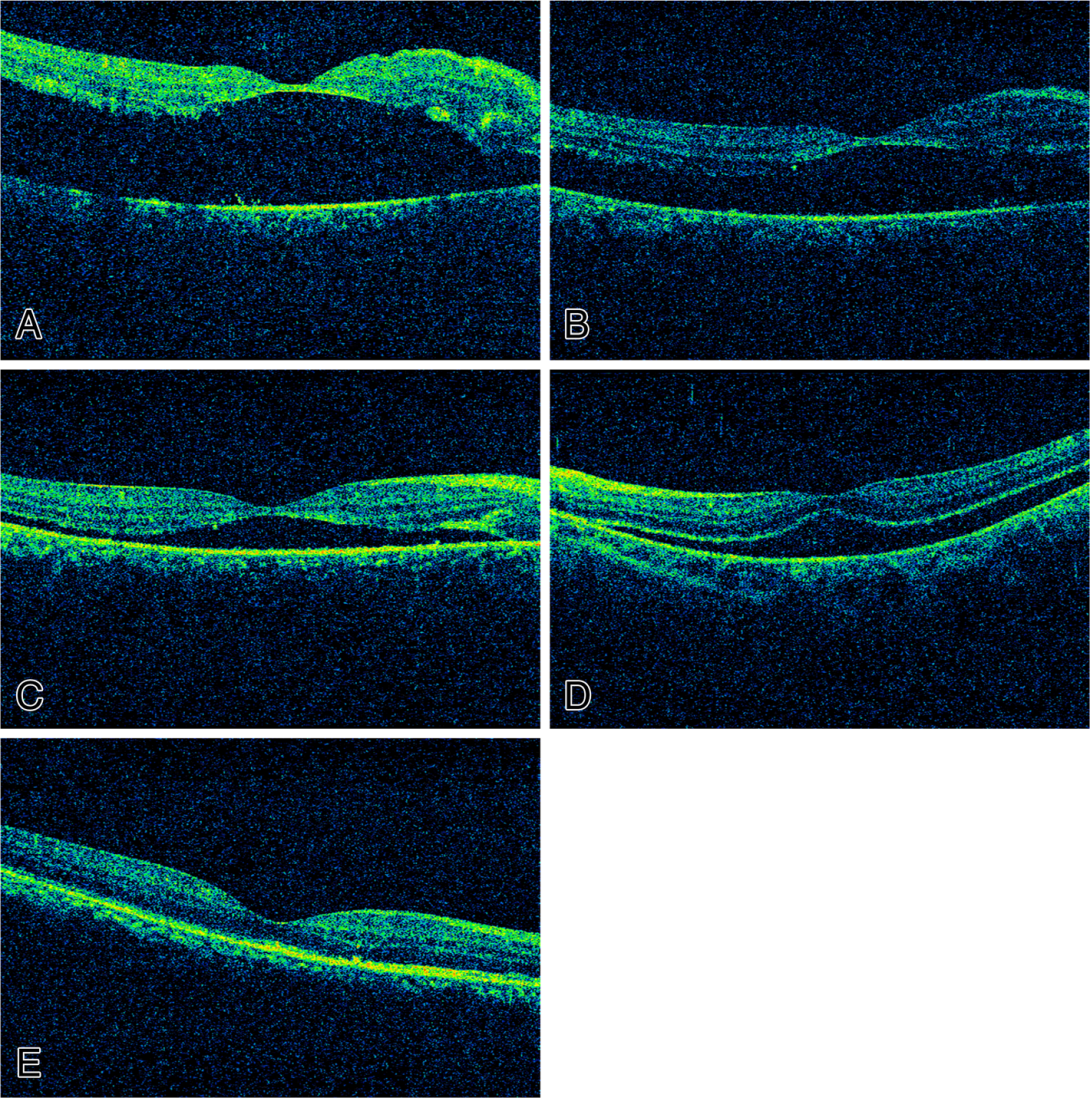
**Optical coherence tomographic images of preoperative and postoperative retinas. A**-**C**: Shows a patient from the SB group. The patient was a 34 year old female, with Poor visual acuity (VA) and metamorphopsia for 15 days before operation. **A**. The best-corrected visual acuity (BCVA) among the preoperative retinas was 2.0. **B**. Typical residual retinal detachment can be seen 1 week after sclera bucking; the BCVA was 1.3. **C**, Typical residual retinal detachment can be seen 2 months after sclera bucking; the BCVA was 1.0. **D**-**E**: Shows a patient from the PPV group. The patient was a 42 years old female, with Poor visual acuity (VA) and metamorphopsia for 21 days before operation. **D**. Optical coherence tomographic images of a preoperative retina; the BCVA was 1.3. **E**. One month after primary vitrectomy operation, macular reattachment was successful in this patient, and no subretinal fluid was present; the BCVA was 0.3.

The improvement in the mean VA was significantly greater in PPV group compared to that in SB group (0.7 ± 0.9 logMAR vs. 0.4 ± 0.8 logMAR, P < 0.05). Meanwhile, as detected by SD-OCT, subretinal fluid was present in 26 of the group 1 eyes (81.3%) and 5 of the group 2 eyes (19.2%) at the final follow-up (2 months postoperation), a difference that was statistically significant (P < 0.05) (Figure 1). Further, epiretinal membranes were observed in 5 of the group 1 eyes (15.6%) and 5 of the group 2 eyes (42.3%). This difference was not statistically significant (P = 1.0). No significant differences were observed between groups in terms of the frequency of discontinued ELM and disruptions of ELM or the inner segment/outer segment (IS/OS) junction (Table 2).

**Table 2:** Preoperative and postoperative OCT findings

**Variable**	**SB (n = 32)**		**PPV (n = 26)**		***P *value**
	**A**	**B**	**A**	**B**	
IS/OS Junction disruption	29	22	24	15	0.37
ELM disruption	21	18	17	9	0.44
ERM	3	5	2	5	1.0
Subretinal fluid	32	26	26	5	<0.0001

A: preoperative; B: postoperative; IS/OS: inner segment/outer segment; ELM: external limiting membrane; ERM: epiretinal membranes; SB = scleral buckling; PPV = pars plana vitrectomy.

## Discussion

Vitrectomy has a short operation time, is a standard procedure, and its success rate is approximately the same as scleral buckling for deep retinal tears and multiple retinal tears at different depths [31]. Furthermore, a better success rate for macular reattachment and earlier recovery of VA has been reported for vitrectomy relative to scleral buckling [32-36]. Based on our preliminary results, the mean VA improvement was greater in PPV group compared to that in SB group. The postoperative visual outcome of macular-off RRD was significantly associated with the integrity of the ELM and the IS/OS junction. In addition, the persistent subretinal fluid (SRF) was directly related to metamorphopsia and poor VA. SD-OCT was introduced for advanced imaging, thus reducing acquisition time and allowing high resolution imaging of macular microstructures [28-30]. SD-OCT allows improved visualization of retinal structures, especially photoreceptor cell layers. With the advent of SD-OCT, the subtle changes of the macula including the integrity of the ELM, the IS/OS junction, the SRF could be well defined. In our present study, both of the two groups showed discontinued ELM, disruptions of IS/OS junction and the existence of the SRF. These findings helped us compare the changes in the macula between the SB group and PPV group. The SD-OCT findings showed the SRF was statistically different in the group 1(81.3%) and group 2 (19.2%).

Persistent SRF could be viewed in the patients who had undergone SB surgery. Several reasons result in the persistent SRF in SB surgery. The mean macular microcirculation blood flows were lower in SB surgery than in PPV surgery. Also, choroidal blood flow has been reported to be less reduced in vitrectomy relative to that in scleral buckling [37]. Further, during PPV surgery, the SRF was exchanged by gas as possible. However, in the SB surgery, the SRF was limited drained out. The silicone oil or perfluoropropane (C3F8) can help the retina reattachment. The hemodynamic change may affect the polarity of the RPE, leading to fluid leakage. Furthermore, inflammation induced by condensation of the scleral buckling may accelerate subretinal fluid accumulation [31]. As such, the presence of persistent subretinal fluid after successful scleral buckling surgery for macular-off RRD may influence the final BCVA or anatomic attachment.

The present study focusses on the macular reattachment and the mean VA improvement of macular-off RRD undergone SB and PPV surgery. It showed that PPV group showed a better mean VA than the scleral buckling group at the final follow-up. The SD-OCT findings demonstrated that a better macular reattachment in the PPV group than the SB group for macular-off RRD.

However, other studies showed that SRF gradually disappeared in most cases within 1 year after surgery [26,27]. The long-term follow-up is necessary in our further study.

The limitations of our study were the short follow-up time, the small sample size, and the subjective nature of the interpretations of the images. In the present study, no attempt was made to determine the long-term complications, such as cataracts and redetachment of the retina. Hence, further follow-up is needed to observe the changes of postoperative VA and SRF.

## Conclusions

SD-OCT contributed to the observation of subtle changes of macula structure of macula-off RRD. SRF that persisted in the SB surgery could influence the VA. We conclude that primary vitrectomy surgery showed advantages in treating macula-off rhegmatogenous retinal detachment. For the macular-off rhegmatogenous retinal detachment patients, PPV surgery helps in the anatomic reduction of the macular and there is no obvious subretinal fluid, thus better visual ability can be achieved.

## Abbreviations

BCVA: Best-corrected visual acuity; ELM: External limiting membrane; logMAR: Logarithm of the minimal angle of resolution; OCT: Optical coherence tomography; SD-OCT: Spectral-domain optical coherence tomography; PPV: Pars plana vitrectomy; RRD: Rhegmatogenous retinal detachment; VA: Visual acuity; SRF: Subretinal fluid.

## Competing interests

The authors declare that they have no competing interests.

## Authors’ contributions

CH, TZ, and TF participated in the design of the study and performed the statistical analysis. CH participated in the sequence alignment and drafted the manuscript. XW and QJ conceived the study, and participated in its design and coordination and helped to draft the manuscript. All authors read and approved the final manuscript.

## Pre-publication history

The pre-publication history for this paper can be accessed here:

http://www.biomedcentral.com/1471-2415/13/12/prepub
